# Early dynamics of eosinophils and the neutrophil-to-lymphocyte ratio are associated with severe immune-related adverse events during nivolumab therapy for metastatic renal cell carcinoma

**DOI:** 10.3389/fimmu.2026.1944728

**Published:** 2026-10-12

**Authors:** Ondřej Fiala, Jan Králíček, Jindřich Kopecký, Michal Vočka, Tomas Buchler, Hana Študentová, Jana Obertová, Beáta Rondzíková, Martin Matějů, Dominik Šafčák, Matej Hrnčár, Peter Priester, Martina Spisarová, Anežka Zemánková, Radka Lohynská, Michaela Tkadlecová, Lucie Grmelová, Petr Stránský, Bohuslav Melichar, Patrik Palacka, Alexandr Poprach

**Affiliations:** 1 Department of Oncology and Radiotherapeutics, Faculty of Medicine and University Hospital in Pilsen, Charles University, Pilsen, Czechia; 2 Biomedical Center, Faculty of Medicine in Pilsen, Charles University, Pilsen, Czechia; 3 ARON Research Foundation ETS, Macerata, Italy; 4 Department of Oncology, University Hospital in Hradec Králové, Hradec Králové, Czechia; 5 Department of Oncology, First Faculty of Medicine, Charles University and General University Hospital, Prague, Czechia; 6 Department of Oncology, Second Faculty of Medicine, Charles University and Motol and Homolka University Hospital, Prague, Czechia; 7 Department of Oncology, Palacký University Medical School and Teaching Hospital, Olomouc, Czechia; 8 2^nd^ Department of Oncology, Faculty of Medicine, Comenius University, and National Cancer Institute, Bratislava, Slovakia; 9 Department of Radiotherapy and Oncology, Faculty of Medicine, Pavol Jozef Šafárik University in Košice, Košice, Slovakia; 10 Department of Oncology, F. D. Roosevelt University General Hospital, Banská Bystrica, Slovakia; 11 Department of Oncology, First Faculty of Medicine, Charles University and Thomayer University Hospital, Prague, Czechia; 12 Department of Comprehensive Cancer Care, Masaryk Memorial Cancer Institute, Brno, Czechia; 13 Department of Comprehensive Cancer Care, Faculty of Medicine, Masaryk University, Brno, Czechia; 14 Department of Urology, Faculty of Medicine and University Hospital in Pilsen, Charles University, Pilsen, Czechia; 15 Cancer Research Institute, Biomedical Research Center of the Slovak Academy of Sciences, Bratislava, Slovakia

**Keywords:** biomarker dynamics, CBC-based immune biomarkers, eosinophils, immune activation, immune-related adverse events, metastatic renal cell carcinoma (mRCC), neutrophil-to-lymphocyte ratio (NLR), nivolumab

## Abstract

**Background:**

Reliable biomarkers for characterization of the risk of severe immune-related adverse events (irAEs) during immune checkpoint inhibition remain lacking. We hypothesized that early dynamics of complementary complete blood count (CBC)-based immune biomarkers may be associated with severe immune toxicity. We therefore evaluated longitudinal changes in absolute eosinophil count (AEC) and neutrophil-to-lymphocyte ratio (NLR) in patients with metastatic renal cell carcinoma (mRCC) treated with nivolumab.

**Methods:**

This multicenter retrospective study included 503 patients with mRCC receiving nivolumab monotherapy in the second or later treatment line. Biomarker analyses were performed using a prespecified 28-day landmark design. AEC and NLR were evaluated at baseline, month 1, and as dynamic Low→High phenotypes, defined as transition from below the baseline upper-quartile threshold to the highest month-1 quartile. Multivariable logistic regression adjusted for age, sex, ECOG performance status, IMDC risk group, and treatment line.

**Results:**

Among 501 toxicity-evaluable patients, 57 (11.4%) developed grade 3–4 irAEs. Baseline AEC and NLR were not associated with severe irAEs. In contrast, the AEC Low→High phenotype was associated with severe irAEs after multivariable adjustment (adjusted odds ratio [aOR] 2.25, 95% confidence interval [CI] 1.02–4.97; P = 0.045), as was the NLR Low→High phenotype (aOR 2.77, 95% CI 1.25–6.17; P = 0.012). In a prespecified combined multivariable model, NLR remained independently associated with severe irAEs (aOR 2.81, 95% CI 1.25–6.30; P = 0.012), whereas the AEC association was attenuated (aOR 2.08, 95% CI 0.90–4.80; P = 0.085).

**Conclusions:**

Early dynamics of eosinophils and NLR, rather than baseline biomarker values, were associated with severe irAEs during nivolumab therapy. These findings suggest that eosinophil expansion and inflammatory remodeling may reflect aspects of early immune activation associated with clinically significant immune toxicity. Longitudinal assessment of routinely available CBC-based immune biomarkers therefore warrants prospective validation in cohorts with time-resolved toxicity assessment.

## Graphical Abstract

**Figure:**
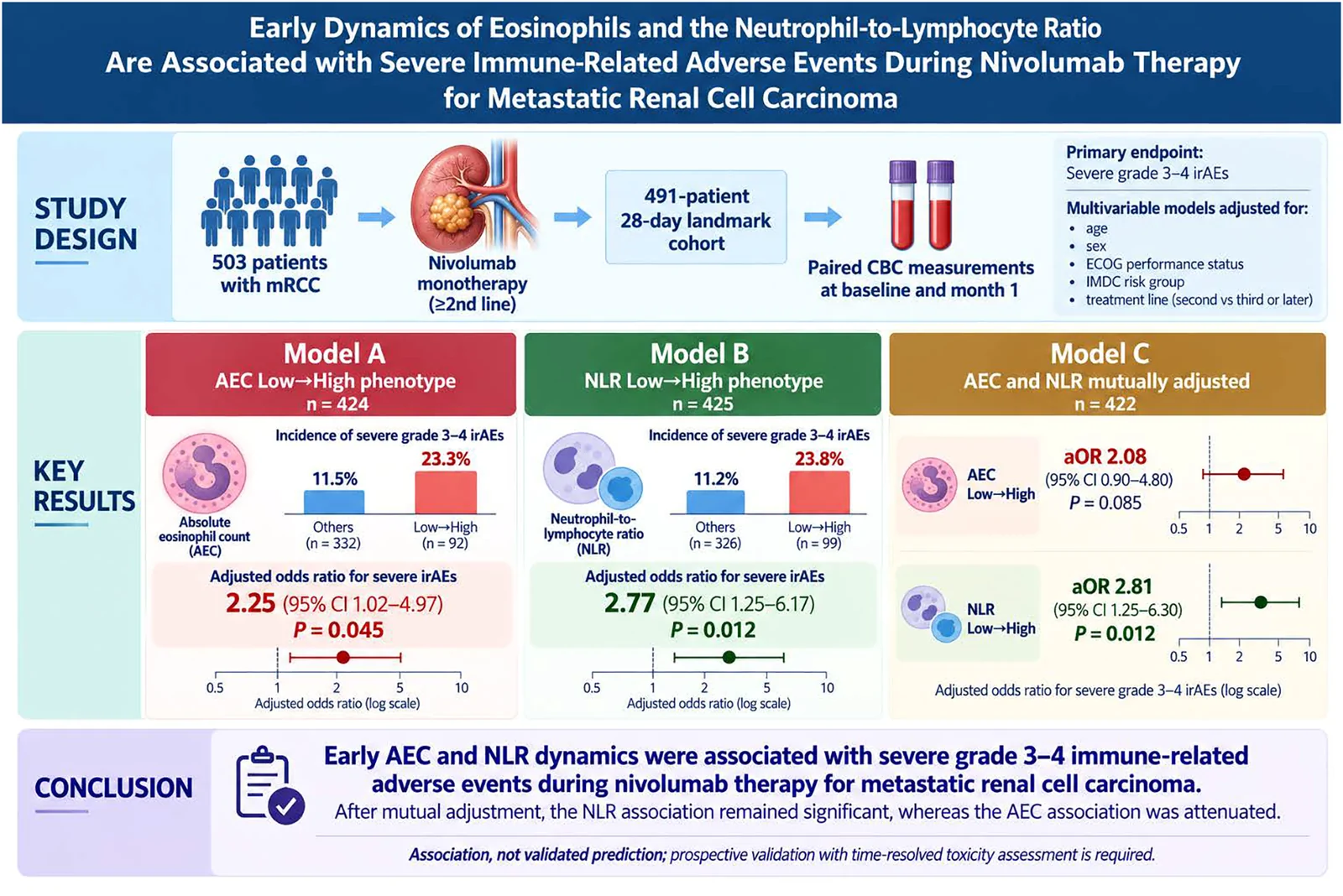

## Introduction

Immune checkpoint inhibitors (ICIs) have substantially improved outcomes in patients with metastatic renal cell carcinoma (mRCC). Although contemporary first-line treatment increasingly relies on combination regimens incorporating ICIs together with VEGF-targeted therapies or dual immune checkpoint blockade, nivolumab monotherapy represented a landmark advance in previously treated mRCC and remains an important model for investigating immune-mediated treatment effects and toxicity mechanisms (1, 2). In the phase III CheckMate 025 trial, nivolumab demonstrated superior overall survival and improved tolerability compared with everolimus in previously treated patients, establishing nivolumab as a standard treatment option in the later-line setting (1, 2). Despite these benefits, ICIs are associated with a distinct spectrum of immune-related adverse events (irAEs), which may affect virtually any organ system and can lead to treatment interruption, hospitalization, prolonged immunosuppressive therapy, or permanent treatment discontinuation (3).

Although most irAEs are manageable, severe grade 3–4 irAEs remain clinically relevant because of their potential morbidity and impact on treatment delivery. Grade 3–4 irAE affect only a relatively small proportion of patients and manifest in different organs, further complicating the efforts to study this important topic. Currently, no widely accepted biomarker is available to identify patients at increased risk of severe irAEs before or shortly after treatment initiation. Most proposed predictors have demonstrated limited reproducibility across studies, while many require specialized testing that is not routinely available in clinical practice. Easily accessible complete blood count (CBC)-based biomarkers therefore represent an attractive strategy for investigating susceptibility to immune-related toxicity. Among routinely available CBC-based inflammatory biomarkers, absolute eosinophil count (AEC) has emerged as a potential indicator of immune activation during ICI therapy, with reported associations with both treatment immune-related toxicity and efficacy across different tumor types (4–9). Another readily available immune biomarker, the neutrophil-to-lymphocyte ratio (NLR), reflects the balance between systemic inflammation and adaptive antitumor immunity and has been associated with clinical outcomes across multiple ICI-treated malignancies, including mRCC (6, 10–12). However, relatively little is known about whether early complementary dynamics of CBC-based immune biomarkers identify patients at increased risk of severe irAEs, particularly within disease-specific cohorts. Given that eosinophil expansion and inflammatory changes likely reflect broader mechanisms of immune activation rather than drug-specific effects, insights derived from nivolumab-treated patients may remain highly relevant to contemporary ICI-based treatment strategies.

We hypothesized that early complementary immune biomarker dynamics may be associated with the occurrence of severe irAEs during nivolumab therapy for mRCC. Therefore, we evaluated baseline values, month-1 values, and early dynamic changes in two prespecified biomarkers, AEC and NLR, in a large multicenter cohort of mRCC patients treated with nivolumab monotherapy. The primary aim of this study was to evaluate the associations between early CBC-based immune biomarker dynamics, represented by AEC and NLR, and severe grade 3–4 irAEs.

## Methods and patients

### Study design and patient population

Patients with mRCC treated with nivolumab monotherapy in the second or later treatment line were retrospectively identified from the multicenter Renal Cell Carcinoma Information System II (RENIS II) registry (http://renis.registry.cz), which has been described previously (13). Baseline demographic, clinicopathological, treatment, laboratory, and toxicity data were extracted from the registry database. The RENIS II registry and the use of registry data for analysis were approved on October 28, 2019 (nr. 201928/52/MOU), by the Multicentre Ethics Committee of the Masaryk Memorial Cancer Institute in Brno, Czech Republic. The study was conducted in accordance with the Declaration of Helsinki and local institutional requirements. An informed consent was signed by all the patients included in the study.

### Study endpoints

The primary endpoint was the occurrence of severe irAEs, defined as grade 3–4 toxicities according to the National Cancer Institute Common Terminology Criteria for Adverse Events (CTCAE), version 5.0. IrAE attribution and grading were performed by the treating investigators and recorded in the registry without central or independent adjudication. Detailed information regarding the exclusion of alternative causes was not systematically available in the registry.

The present study evaluated the associations between two prespecified CBC-based biomarkers, AEC and NLR, and the risk of severe irAEs. Biomarkers were evaluated using both static (baseline and month-1) and dynamic (Low→High trajectory) approaches. Multivariable logistic regression models were subsequently used to assess the independent associations of AEC and NLR with severe irAE risk after adjustment for prespecified clinically relevant covariates.

As an exploratory analysis, an ordinal CBC-based immune biomarker activation score was constructed according to the number of positive dynamic biomarker phenotypes (0 = neither AEC nor NLR Low→High; 1 = either phenotype; 2 = both phenotypes) to evaluate whether increasing activation of complementary CBC-based immune biomarkers was associated with severe irAE risk.

### Landmark cohort

To minimize guarantee-time bias when evaluating early biomarker dynamics, all biomarker analyses were performed within a prespecified 28-day landmark framework. Patients who did not reach the prespecified 28-day landmark, irrespective of the reason (e.g., death or insufficient follow-up), were excluded from biomarker analyses because they were not eligible for longitudinal assessment of early biomarker changes and had limited opportunity for subsequent manifestation of severe immune-related adverse events. All biomarker analyses, including baseline biomarker analyses, were therefore performed within the landmark cohort. Dynamic biomarker analyses additionally required evaluable biomarker measurements at both baseline and approximately one month after treatment initiation. The landmark criterion was applied solely to define eligibility for biomarker analyses and did not modify the study endpoint. Severe grade 3–4 irAEs were evaluated irrespective of their timing during follow-up because the exact dates of irAE onset were not available in the registry.

### Biomarker assessment and definitions

AEC and NLR were prespecified peripheral immune biomarkers selected to evaluate complementary aspects of systemic immune activation during nivolumab treatment. AEC was assessed at baseline and approximately one month after nivolumab initiation. Baseline AEC was defined as the measurement obtained closest to treatment initiation before the first nivolumab administration. NLR was calculated as the ratio of the absolute neutrophil count to the absolute lymphocyte count using laboratory measurements obtained at the same baseline and month-1 time points.

To evaluate both static and dynamic biomarker associations, AEC and NLR values were categorized using cohort-specific upper-quartile (Q4) cut-offs derived separately for baseline and month-1 measurements within the evaluable landmark population. Patients in the highest quartile (Q4) were compared with those in quartiles 1–3 (Q1–3).

To characterize early biomarker dynamics, trajectory phenotypes were defined for both biomarkers. The AEC Low→High phenotype was defined as a transition from below the baseline Q4 threshold to within the month-1 Q4. Analogously, the NLR Low→High phenotype was defined as a transition from below the baseline Q4 threshold to within the month-1 Q4. Patients remaining within Q1–3 or Q4 at both time points, or transitioning from Q4 at baseline to Q1–3 at month 1, were classified as non-Low→High. The Low→High trajectory was prespecified to identify patients exhibiting a marked upward shift into the upper range of the biomarker distribution between baseline and month 1. Patients remaining within Q1–3 or Q4 at both time points, or transitioning from Q4 at baseline to Q1–3 at month 1, constituted the reference group. Cohort-specific Q4 cut-offs were derived separately for baseline and month-1 measurements within the respective evaluable landmark populations. The Q4 cut-offs were 0.230 ×10^9^/L at baseline and 0.298 ×10^9^/L at month 1 for AEC, and 4.154 at baseline and 4.534 at month 1 for NLR. These thresholds were used as analytical cut-offs and should not be interpreted as externally validated clinical thresholds.

### Statistical analyses

Continuous variables are presented as medians with interquartile ranges (IQRs), whereas categorical variables are presented as frequencies and percentages. Follow-up duration was estimated using the reverse Kaplan–Meier method, in which deaths were treated as censored observations and patients remaining alive at last contact were considered events. Median follow-up time is reported with corresponding 95% confidence intervals (CIs). Baseline characteristics were compared between patients with and without severe irAEs using the Mann–Whitney U test for continuous variables and the χ² test or Fisher’s exact test for categorical variables, as appropriate. Complete-case analyses were performed for each biomarker analysis; therefore, evaluable sample sizes differed according to the availability of baseline and month-1 laboratory measurements.

Associations between biomarker categories and severe irAE occurrence were initially evaluated using univariable logistic regression models. Odds ratios (ORs) with corresponding 95% CIs were estimated for baseline biomarker status, month-1 biomarker status, and dynamic Low→High phenotypes for both AEC and NLR. Prespecified multivariable logistic regression models were used to evaluate the associations between dynamic biomarker phenotypes and severe irAEs after adjustment for clinically relevant covariates, including age, sex, ECOG performance status (≥1 vs. 0), IMDC risk group (poor vs. favorable/intermediate), and treatment line (second vs. third or later). Separate models were constructed for the AEC Low→High phenotype and the NLR Low→High phenotype. An additional prespecified multivariable logistic regression model simultaneously incorporated both the AEC Low→High and NLR Low→High phenotypes together with the same clinical covariates to evaluate their mutually adjusted associations with severe irAE risk.

An exploratory immune biomarker activation score (0–2) was defined as the number of positive dynamic biomarker phenotypes and was evaluated descriptively.

Sensitivity analyses were performed to assess the robustness of the primary quartile-based findings. First, AEC and NLR changes were evaluated as continuous variables, expressed as absolute change (month 1 minus baseline) and percentage change relative to baseline. Absolute AEC change was modeled per 0.1 ×10^9^/L increase and absolute NLR change per 1-unit increase; percentage changes were modeled per 100% increase. Continuous-change models were adjusted for the same prespecified clinical covariates as the primary multivariable analyses, including age, sex, ECOG performance status, IMDC risk group, and treatment line. Second, Low→High phenotypes were redefined using median and upper-tertile thresholds to assess the sensitivity of the findings to the prespecified Q4 cut-off. Third, because of the limited number of severe irAE events among patients with Low→High phenotypes, the principal multivariable analyses were repeated using Firth penalized logistic regression. Finally, baseline clinical characteristics and severe irAE incidence were compared between patients with complete paired AEC and NLR measurements and landmark-eligible patients without complete paired biomarker data to assess potential selection bias.

All statistical tests were two-sided, and p values <0.05 were considered statistically significant. Data management, cohort derivation, statistical analyses, quality-control procedures, reproducibility analyses, and figure generation were performed using Python version 3.12 (Python Software Foundation, Wilmington, DE, USA). Reverse Kaplan–Meier estimation of follow-up duration was independently verified using R version 4.3.2 (R Foundation for Statistical Computing, Vienna, Austria). All cohort derivations, biomarker definitions, statistical analyses, tables, and figures were generated using a predefined reproducible analytical workflow incorporating automated quality-control procedures and patient-level cohort verification.

## Results

### Patients

A total of 503 patients with mRCC treated with nivolumab monotherapy were included in the overall study population. Of the 503 patients included in the overall cohort, 501 had evaluable toxicity information and 491 reached the prespecified 28-day landmark for biomarker analyses. Severe grade 3–4 irAEs occurred in 57/501 (11.4%) patients, whereas toxicity information was unavailable for two patients. The median follow-up was 35.8 months (95% CI, 32.9–40.5 months), as estimated using the reverse Kaplan–Meier method. A total of 491 patients reached the prespecified 28-day landmark and constituted the landmark biomarker cohort used for all subsequent biomarker analyses. Individual analyses were subsequently performed in evaluable subsets according to the availability of baseline and month-1 laboratory measurements. The derivation of the landmark cohort and biomarker-specific analytical subsets is summarized in **Supplementary Figure S1**, whereas baseline patient characteristics are presented in **Table 1**. Among the 491 landmark-eligible patients with known toxicity status, 422 had complete paired AEC and NLR measurements. Severe irAEs occurred in 53/422 (12.6%) patients with complete paired measurements and in 3/69 (4.3%) patients without complete paired biomarker data (p=0.063).

**Table 1:** Baseline characteristics of the overall study population and the landmark biomarker cohort.

Characteristic	Overall cohort (n = 503)	Landmark biomarker cohort (n = 491)
Patients, n	503	491
Age, years, median (IQR)	67.4 (60.6–72.9)	67.6 (60.6–73.0)
Sex, n (%)		
Male	370 (73.6)	360 (73.3)
Female	133 (26.4)	131 (26.7)
Histology, n (%)		
Clear-cell RCC	337 (67.0)	330 (67.2)
Non-clear-cell RCC	32 (6.4)	31 (6.3)
Unknown	134 (26.6)	130 (26.5)
Tumor grade, n (%)		
G1–2	193 (38.4)	190 (38.7)
G3–4	229 (45.5)	223 (45.4)
Unknown	81 (16.1)	78 (15.9)
Metastatic disease at diagnosis, n (%)		
Synchronous	189 (37.6)	181 (36.9)
Metachronous	286 (56.9)	282 (57.4)
Unknown	28 (5.6)	28 (5.7)
Previous nephrectomy, n (%)		
Yes	413 (82.1)	407 (82.9)
No	90 (17.9)	84 (17.1)
ECOG performance status, n (%)		
0	154 (30.6)	154 (31.4)
≥1	314 (62.4)	305 (62.1)
Unknown	35 (7.0)	32 (6.5)
IMDC risk group, n (%)		
Favorable	134 (26.6)	133 (27.1)
Intermediate	248 (49.3)	243 (49.5)
Poor	57 (11.3)	52 (10.6)
Unknown	64 (12.7)	63 (12.8)
Treatment line, n (%)		
Second line	312 (62.0)	304 (61.9)
Third or later line	189 (37.6)	187 (38.1)
Unknown	2 (0.4)	0 (0.0)
Baseline absolute eosinophil count (×10^9^/L), median (IQR)	0.13 (0.08–0.23)	0.14 (0.08–0.23)
Baseline neutrophil-to-lymphocyte ratio, median (IQR)	2.84 (1.85–4.21)	2.81 (1.85–4.15)
Median follow-up, months (95% CI)	35.8 (32.9–40.5)	35.8 (32.9–40.5)
Patients with severe grade 3–4 irAEs, n (%)	57 (11.4)	56 (11.4)

ECOG, Eastern Cooperative Oncology Group; IMDC, International Metastatic RCC Database Consortium; RCC, renal cell carcinoma; irAE, immune-related adverse events; L, liter; IQR, interquartile range; CI, confidence interval.

The landmark biomarker cohort comprised patients who reached the prespecified 28-day landmark and were therefore eligible for biomarker analyses. Continuous variables are presented as median (IQR) and categorical variables as number (%).

### Absolute eosinophil count

Baseline AEC was not associated with severe grade 3–4 irAEs (**Table 2**). In contrast, patients in the highest month-1 AEC quartile had significantly increased odds of severe irAEs compared with those in quartiles 1–3 (OR 1.93, 95% CI 1.06–3.53; p = 0.032). Similarly, patients exhibiting the AEC Low→High phenotype experienced severe irAEs more frequently than those without this phenotype (23.3% vs. 11.5%) and had more than twofold higher odds of severe toxicity (OR 2.32, 95% CI 1.07–5.03; p = 0.033). After adjustment for age, sex, ECOG performance status, IMDC risk group, and treatment line (Model A), the AEC Low→High phenotype remained independently associated with severe irAEs (adjusted OR 2.25, 95% CI 1.02–4.97; p = 0.045) (**Table 3**; **Figure 1**).

**Table 2:** Associations of prespecified immune biomarkers with severe grade 3–4 immune-related adverse events (irAEs) in the 28-day landmark cohort.

Biomarker	Analysis	Comparison	Severe irAEs/total (%)	OR (95% CI)	*p-value*
AEC	Baseline	Q4 vs Q1–3	16/113 (14.2) vs 38/333 (11.4)	1.28 (0.68–2.40)	0.440
	Month 1	Q4 vs Q1–3	20/107 (18.7) vs 34/320 (10.6)	1.93 (1.06–3.53)	0.032
	Dynamics	Low→High vs others	10/43 (23.3) vs 44/381 (11.5)	2.32 (1.07–5.03)	0.033
NLR	Baseline	Q4 vs Q1–3	13/113 (11.5) vs 41/337 (12.2)	0.94 (0.48–1.82)	0.851
	Month 1	Q4 vs Q1–3	18/107 (16.8) vs 35/319 (11.0)	1.64 (0.89–3.04)	0.115
	Dynamics	Low→High vs others	10/42 (23.8) vs 43/383 (11.2)	2.47 (1.14–5.38)	0.023

OR, odds ratio; CI, confidence interval; AEC, absolute eosinophil count; NLR, neutrophil-to-lymphocyte ratio; irAE, immune-related adverse events.

Odds ratios (ORs) and corresponding 95% confidence intervals (CIs) were estimated using univariable logistic regression. Quartile analyses compared patients in the highest quartile (Q4) with those in quartiles 1–3 (Q1–3), with quartile cut-offs derived separately for baseline and month-1 measurements within the landmark cohort. The Low→High phenotype was defined as a transition from below the baseline Q4 threshold to within the month-1 Q4.

**Table 3:** Multivariable logistic regression models evaluating associations between early AEC and NLR dynamics and severe grade 3–4 immune-related adverse events (irAEs).

Model A. AEC model		
Variable	aOR (95% CI)	p-value
AEC Low→High phenotype	2.25 (1.02–4.97)	0.045
Age, per year	1.03 (1.00–1.06)	0.078
Male sex	0.89 (0.47–1.71)	0.730
ECOG PS ≥1	1.01 (0.54–1.86)	0.984
IMDC poor risk	0.44 (0.13–1.49)	0.187
Third or later treatment line	0.81 (0.43–1.51)	0.499
Model B. NLR model		
Variable	aOR (95% CI)	p-value
NLR Low→High phenotype	2.77 (1.25–6.17)	0.012
Age, per year	1.03 (1.00–1.07)	0.061
Male sex	0.90 (0.47–1.72)	0.743
ECOG PS ≥1	0.93 (0.50–1.74)	0.830
IMDC poor risk	0.43 (0.13–1.46)	0.176
Third or later treatment line	0.76 (0.41–1.43)	0.397
Model C. Combined CBC-based immune biomarker model		
Variable	aOR (95% CI)	p-value
AEC Low→High phenotype	2.08 (0.90–4.80)	0.085
NLR Low→High phenotype	2.81 (1.25–6.30)	0.012
Age, per year	1.03 (1.00–1.07)	0.063
Male sex	0.85 (0.44–1.64)	0.626
ECOG PS ≥1	0.91 (0.49–1.70)	0.765
IMDC poor risk	0.43 (0.13–1.46)	0.175
Third or later treatment line	0.80 (0.42–1.50)	0.488

aOR, adjusted odds ratio; CI, confidence interval; AEC, absolute eosinophil count; NLR, neutrophil-to-lymphocyte ratio; ECOG PS, Eastern Cooperative Oncology Group Performance Status; IMDC, International Metastatic RCC Database Consortium; CBC, complete blood count.

Model A included 424 patients (54 severe irAEs), Model B included 425 patients (53 severe irAEs), and Model C included 422 patients (53 severe irAEs).

All multivariable models were adjusted for age, sex, ECOG performance status (≥1 vs 0), IMDC poor-risk group, and treatment line (second vs third or later). Model A evaluated the AEC Low→High phenotype, Model B evaluated the NLR Low→High phenotype, and Model C simultaneously included both prespecified biomarker phenotypes.

**Figure 1:**
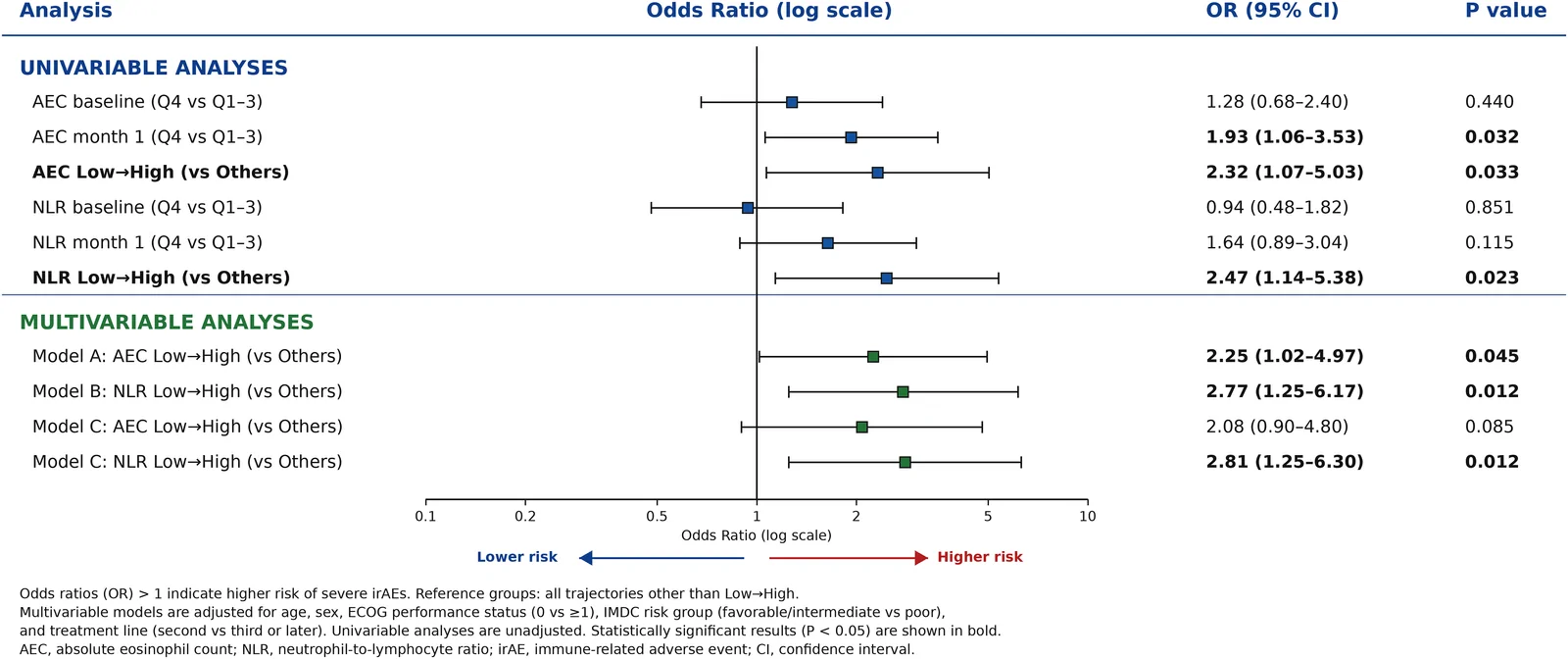
Associations between early AEC and NLR dynamics and severe grade 3–4 immune-related adverse events (irAEs). Forest plot showing univariable and multivariable associations of baseline, month-1, and dynamic AEC and NLR measures with severe grade 3–4 immune-related adverse events (irAEs). Odds ratios (ORs) >1 indicate a higher risk of severe irAEs. Reference groups for the dynamic analyses comprised all trajectories other than Low→High. Models A and B evaluated AEC and NLR Low→High phenotypes separately, whereas Model C simultaneously included both biomarker phenotypes. Multivariable models were adjusted for age, sex, ECOG performance status (≥1 vs 0), IMDC poor-risk group, and treatment line (second vs third or later). Univariable analyses were unadjusted. Statistically significant results (p<0.05) are shown in bold. AEC, absolute eosinophil count; NLR, neutrophil-to-lymphocyte ratio; irAE, immune-related adverse event; OR, odds ratio; CI, confidence interval; ECOG, Eastern Cooperative Oncology Group; IMDC, International Metastatic Renal Cell Carcinoma Database Consortium.

### Neutrophil-to-lymphocyte ratio

Neither baseline nor month-1 NLR demonstrated statistically significant associations with severe irAEs in quartile-based analyses (**Table 2**). However, patients exhibiting the NLR Low→High phenotype likewise experienced severe irAEs more frequently than those without this phenotype (23.8% vs. 11.2%) and had significantly increased odds of severe toxicity (OR 2.47, 95% CI 1.14–5.38; p = 0.023). After adjustment for the same clinical covariates (Model B), the NLR Low→High phenotype remained independently associated with severe irAE risk (adjusted OR 2.77, 95% CI 1.25–6.17; p = 0.012) (**Table 3**; **Figure 1**).

### Combined analysis of AEC and NLR

To evaluate their mutually adjusted associations with severe irAE risk, both biomarker trajectories were simultaneously included in a prespecified multivariable model (Model C). Within this model, the NLR Low→High phenotype remained independently associated with severe irAE risk (adjusted OR 2.81, 95% CI 1.25–6.30; p = 0.012), whereas the association for the AEC Low→High phenotype was attenuated but remained of similar magnitude (adjusted OR 2.08, 95% CI 0.90–4.80; p = 0.085) (**Table 3**; **Figure 1**).

### Composite inflammatory activation phenotype

To explore the occurrence of severe irAEs according to the number of positive dynamic biomarker phenotypes, we evaluated an exploratory biomarker activation score defined as the number of positive dynamic biomarker phenotypes (0 = neither AEC Low→High nor NLR Low→High, 1 = either phenotype, 2 = both phenotypes). The incidence of severe grade 3–4 irAEs was 10.9% in patients with score 0 to 16.7% in those with score 1 and 100% in the three patients with score 2 (**Supplementary Table S1**).

Given the very small number of patients with score 2, these findings are presented descriptively and should be interpreted with caution.

### Sensitivity analyses

When biomarker changes were evaluated continuously, the median absolute AEC change was 0.035 ×10^9^/L among patients with severe irAEs and 0.030 ×10^9^/L among those without severe irAEs. Greater absolute AEC increase was associated with severe irAEs after multivariable adjustment (aOR 1.13 per 0.1 ×10^9^/L increase, 95% CI 1.02–1.25; p=0.016), whereas percentage AEC change was not (aOR 1.03 per 100% increase; p=0.700). Median absolute NLR changes were 0.375 and 0.324, respectively. Neither absolute NLR change (aOR 1.10 per 1-unit increase, 95% CI 0.98–1.24; p=0.102) nor percentage NLR change (aOR 1.15 per 100% increase; p=0.412) was significantly associated with severe irAEs when modeled continuously (**Supplementary Table S2**).

Sensitivity analyses using alternative thresholds suggested that the associations were more apparent for pronounced upward biomarker transitions. Median-based Low→High phenotypes were not associated with severe irAEs for either AEC or NLR. Using upper-tertile thresholds, effect estimates were similar to those obtained with the primary Q4 definition for AEC (aOR 2.27, p=0.053) and NLR (aOR 2.67, p=0.012), compared with aORs of 2.25 (p=0.045) and 2.77 (p=0.012), respectively, using Q4 thresholds (**Supplementary Table S3**). When Q4-based dynamics were further examined across all four possible trajectories, increased severe irAE risk was observed specifically in the Low→High groups. Neither persistently high (High→High) nor decreasing (High→Low) trajectories were significantly associated with severe irAEs compared with the Low→Low reference group for either AEC or NLR (**Supplementary Table S4**).

Because only 10 severe irAEs occurred within each Low→High subgroup, the primary multivariable analyses were repeated using Firth penalized logistic regression. Effect estimates remained similar to those obtained with conventional logistic regression for both AEC (OR 2.28, 95% CI 1.05–4.96; p=0.038) and NLR (OR 2.78, 95% CI 1.27–6.09; p=0.011), supporting the robustness of the primary associations despite the limited number of events (**Supplementary Table S5**).

Finally, among 491 landmark-eligible patients with known toxicity status, 422 had complete paired AEC and NLR measurements. Severe irAEs occurred in 53/422 (12.6%) patients with complete paired measurements and in 3/69 (4.3%) patients without complete paired biomarker data (p=0.063). Patients with complete paired measurements also differed from those without complete data with respect to ECOG performance status and IMDC risk group (**Supplementary Table S6**).

### Spectrum of severe irAEs

The spectrum of severe grade 3–4 irAEs was broadly distributed across organ systems and did not suggest that the associations of AEC or NLR dynamics with severe irAE risk were driven by a single toxicity type. Hepatic, renal, pulmonary, neurologic, rheumatologic/musculoskeletal, dermatologic, gastrointestinal, and endocrine/metabolic toxicities were observed across biomarker-defined subgroups. No individual toxicity category was significantly enriched according to either the AEC Low→High or NLR Low→High phenotype (all Fisher’s exact p > 0.05; **Supplementary Table S7**).

## Discussion

The present multicenter study demonstrates that early dynamics of routinely available CBC-based immune biomarkers are associated with severe grade 3–4 irAEs during nivolumab therapy for mRCC. When both dynamic phenotypes were evaluated simultaneously, the association remained statistically significant for NLR, whereas the AEC association was attenuated, suggesting that these biomarkers may capture partially overlapping components of the evolving immune response during PD-1 blockade. Collectively, these findings support further investigation of dynamic rather than static CBC-based immune biomarker assessment in relation to immune toxicity. More broadly, they are consistent with the concept that immune activation during treatment may simultaneously promote antitumor efficacy and increase susceptibility to immune-related toxicity (14–17).

The role of eosinophils in immune activation is supported by accumulating experimental and clinical evidence. Eosinophils actively participate in innate and adaptive immune responses through cytokine secretion, antigen presentation, and interactions with activated T lymphocytes (18–21). Experimental studies have demonstrated that eosinophils promote CD8^+^ T-cell recruitment into the tumor microenvironment, thereby enhancing antitumor immunity and providing a biological basis for their association with immune-related toxicity (22). Consistent with these biological observations, several clinical studies have associated eosinophil-based biomarkers with both improved treatment outcomes and immune-related toxicity during immune checkpoint inhibition (4–9). In the present study, eosinophil dynamics, but not baseline eosinophil levels, were associated with clinically relevant grade 3–4 irAEs, with the association also observed when absolute AEC change was modeled continuously.

Unlike eosinophil counts, the NLR reflects the balance between neutrophil-driven systemic inflammation and lymphocyte-mediated adaptive immunity. Neutrophils contribute to immune regulation through secretion of cytokines, chemokines, reactive oxygen species, and other mediators that influence T-cell activation, whereas lymphocytes reflect the capacity to mount effective antitumor immune responses (23). Consequently, early NLR dynamics may reflect remodeling of systemic immune homeostasis following PD-1 blockade rather than simply pre-existing inflammatory status. Although NLR has traditionally been investigated as a prognostic biomarker in oncology (23), increasing evidence suggests that early NLR dynamics during immune checkpoint inhibition may also mirror treatment-induced immune activation and immune dysregulation (10, 11). In our sensitivity analyses, however, NLR change was not significantly associated with severe irAEs when modeled as a continuous variable, suggesting that the observed association may not follow a simple linear relationship across the full range of NLR changes.

The relationship between AEC and NLR dynamics also warrants cautious interpretation. Although both biomarkers were individually associated with severe irAEs, simultaneous inclusion in the combined model attenuated the AEC association (aOR 2.08, p=0.085), whereas NLR remained independently associated with severe toxicity. This finding argues against interpreting the two biomarkers as fully independent or non-redundant signals. Rather, they may capture partially overlapping aspects of systemic immune activation, while retaining biologically distinct features: eosinophils participate directly in immune effector mechanisms, whereas NLR reflects the balance between systemic inflammatory activity and adaptive immunity. Further studies are required to determine whether their combined assessment provides clinically meaningful information beyond either biomarker alone.

Present findings should also be interpreted in the context of previous studies evaluating inflammatory biomarkers during immune checkpoint inhibition. Although numerous studies have investigated baseline inflammatory biomarkers, evidence regarding early biomarker dynamics remains comparatively limited. Among the few available studies addressing early biomarker dynamics, Tasaki et al. recently demonstrated that increased eosinophil proportion two weeks after initiation of ipilimumab plus nivolumab, but not baseline eosinophil levels or baseline NLR, was independently associated with severe irAEs in patients with mRCC (8). Despite important methodological differences, including evaluation of eosinophil proportion rather than AEC, combination immune checkpoint blockade, and the absence of a landmark design, their findings further support the potential relevance of early biomarker dynamics compared with isolated baseline measurements. Dynamic NLR assessment has likewise been investigated in mRCC by Lalani et al., who demonstrated that on-treatment changes in NLR were associated with treatment outcomes during immune checkpoint blockade, although their study did not evaluate immune-related toxicity (10). In contrast, Zhang et al., in a recent systematic review and meta-analysis, reported that higher AEC and lower NLR were associated with an increased overall risk of irAEs across heterogeneous tumor types and ICI regimens (6). The apparently opposite NLR direction compared with Zhang et al. may partly reflect the distinction between static NLR levels and dynamic upward transitions. Zhang et al. predominantly evaluated baseline or single-time-point NLR measurements, whereas in the present study baseline NLR was not associated with severe irAEs and the signal was observed specifically among patients undergoing pronounced upward transitions. Continuous NLR change was not significantly associated with severe irAEs, further suggesting that the relationship may not be linear across the full range of NLR changes. Overall, the available evidence supports a biological role of eosinophils in irAE susceptibility. Likewise, the discrepant findings for NLR may reflect important methodological differences. Moreover, Zhang et al. evaluated predominantly static biomarkers and irAEs of all grades across multiple malignancies, whereas the present study focused on early biomarker dynamics in a homogeneous cohort of nivolumab-treated patients with mRCC and exclusively on clinically significant grade 3–4 irAEs. Interpretation of baseline NLR is further complicated by its well-established adverse prognostic value. Patients with elevated baseline NLR generally have shorter exposure to immune checkpoint inhibition because of earlier disease progression or death, potentially reducing the opportunity for immune-related adverse events to develop. The additional sensitivity analyses further refine this interpretation. Absolute AEC change remained associated with severe irAEs when modeled continuously, supporting an association that is not dependent solely on the Q4 categorization. In contrast, continuous NLR change was not statistically significant, whereas transitions into either the upper tertile or Q4 were associated with severe irAEs and median-based transitions were not. Moreover, analysis of all four Q4-based trajectories showed that the increased risk was observed specifically among patients transitioning from Low to High, whereas persistently high and decreasing trajectories were not significantly associated with severe irAEs. Together, these findings suggest that the relationship between biomarker dynamics and severe toxicity may differ between AEC and NLR, with the NLR association being more apparent among pronounced upward transitions rather than across the full continuum of change. Collectively, these observations support the concept that dynamic CBC-based biomarker assessment may provide information beyond that obtained from isolated baseline measurements. The present cohort partially overlaps with our previous registry-based studies (9, 11, 12), which focused primarily on treatment efficacy and related prognostic or predictive factors, including CBC-derived biomarkers. None of these studies evaluated associations between CBC biomarkers and immune-related toxicity, which represents the distinct endpoint of the present analysis.

We did not observe significant associations between either AEC or NLR dynamics and individual categories of severe irAEs. Instead, both biomarkers appeared to reflect a generalized predisposition to severe immune toxicity irrespective of the affected organ system. This finding supports the hypothesis that early systemic immune activation represents a shared biological mechanism underlying susceptibility to severe irAEs rather than determining organ-specific toxicity patterns.

From a clinical perspective, AEC and NLR are inexpensive and universally available laboratory parameters routinely measured during standard medical care, making their longitudinal assessment readily feasible. However, because the temporal relationship between month-1 biomarker measurements and severe irAE onset could not be established in the present dataset, these findings should not be interpreted as demonstrating prospective prediction of toxicity. The observed associations should currently be regarded as hypothesis-generating and require prospective external validation with time-resolved toxicity assessment before any role in clinical risk stratification or treatment modification can be considered.

The strengths of the present study include the relatively large multicenter real-world cohort, the homogeneous population of patients receiving nivolumab monotherapy, the prespecified landmark design minimizing guarantee-time bias, and comprehensive evaluation of both static and dynamic biomarker approaches. Parallel evaluation of AEC and NLR dynamics further enabled assessment of potentially overlapping but biologically distinct dimensions of systemic immune and inflammatory activity using routinely available CBC-derived laboratory parameters. The consistency of the primary associations in Firth penalized regression and the evaluation of continuous changes, alternative thresholds, and individual biomarker trajectories further allowed assessment of the robustness and functional form of the observed associations.

Several limitations should be acknowledged. First, the retrospective observational design introduces the possibility of residual confounding despite multivariable adjustment. Information on intercurrent infections, inflammatory conditions, concomitant medications, and the timing of corticosteroid administration relative to month-1 biomarker assessment was not systematically available, although these factors may influence both AEC and NLR. Second, exact dates of irAE onset were unavailable. Consequently, we could not establish whether month-1 biomarker measurements preceded severe toxicity in individual patients, perform a post-landmark toxicity analysis, or exclude the possibility that an irAE itself or its treatment influenced the observed biomarker change. The present findings therefore demonstrate associations rather than prospective prediction or temporal causality. In addition, irAE attribution and grading were performed by treating investigators without central or independent adjudication, and detailed information regarding the exclusion of competing causes was unavailable. Therefore, some degree of outcome misclassification cannot be excluded. Third, biomarker analyses were restricted to patients with available longitudinal laboratory measurements. Patients with complete paired AEC and NLR measurements differed from those without complete paired data with respect to ECOG performance status and IMDC risk group, while severe irAEs were numerically more frequent in the complete-case group, and selection bias therefore cannot be excluded. The relatively small number of severe irAEs, including only 10 events within each primary Low→High subgroup, limited statistical power for subgroup analyses and the complexity of multivariable modeling, although Firth penalized analyses yielded similar estimates. Finally, the cohort consisted of patients receiving second- or later-line nivolumab monotherapy and therefore does not directly represent contemporary first-line ICI-based combination regimens, in which biomarker dynamics and toxicity profiles may differ. Prospective validation in independent contemporary cohorts with time-resolved toxicity and treatment data is required.

## Conclusions

Early AEC and NLR dynamics were associated with severe grade 3–4 irAEs in patients with mRCC treated with nivolumab, whereas baseline biomarker measurements were not. These findings support further investigation of longitudinal CBC-based biomarker dynamics in relation to immune toxicity. Prospective validation in independent cohorts with time-resolved toxicity assessment is required before their predictive or clinical utility can be established.

## Data Availability

The datasets generated and/or analyzed during the current study are not publicly available due to patient data security but are available from the corresponding author on reasonable request.
