# Supplementary material for: Early dynamics of eosinophils and the neutrophil-to-lymphocyte ratio are associated with severe immune-related adverse events during nivolumab therapy for metastatic renal cell carcinoma
Source: Front Immunol. 2026 Oct 12;17:1944728. doi: 10.3389/fimmu.2026.1944728 (PMC13696394; doi:10.3389/fimmu.2026.1944728)
Supplement: Supplementary file 1 [file DataSheet1.pdf]

Supplementary Data

Early Dynamics of Eosinophils and the Neutrophil-to-Lymphocyte Ratio Are Associated with Severe Immune-Related Adverse Events During Nivolumab Therapy for Metastatic Renal Cell Carcinoma

Ondřej Fiala[1,2,3§], Jan Králíček[1], Jindřich Kopecký[4], Michal Vočka[5], Tomas Buchler[6] Hana Študentová[7], Jana Obertová[8], Beáta Rondzíková[8], Martin Matějů[5], Dominik Šafčák[9], Matej Hrnčár[10], Peter Priester[4], Martina Spisarová[7], Anežka Zemánková[7], Radka Lohynská[11], Michaela Tkadlecová[1], Lucie Grmelová[12,13], Petr Stránský jr.[14], Bohuslav Melichar[7], Patrik Palacka[8,15#], Alexandr Poprach[12,13#]

[1]Department of Oncology and Radiotherapeutics, Faculty of Medicine and University Hospital in Pilsen, Charles University, Czech Republic , alej Svobody 80, 304 60 Pilsen, Czech Republic

[2]Biomedical Center, Faculty of Medicine in Pilsen, Charles University, alej Svobody 76, 304 60 Pilsen, Czech Republic

[3]ARON Research Foundation ETS, Macerata, Italy

[4]Department of Oncology, University Hospital in Hradec Králové, Sokolská 581, Hradec Králové 50005, Czech Republic

[5]Department of Oncology, First Faculty of Medicine , Charles University and General University Hospital, U Nemocnice 499/2, 128 08 Prague, Czech Republic

[6]Department of Oncology, Second Faculty of Medicine, Charles University and Motol and Homolka University Hospital, V Úvalu 84, 150 06 Prague, Czech Republic

[7]Department of Oncology, Palacký University Medical School and Teaching Hospital, I.P. Pavlova 6, Olomouc, 775 20, Czech Republic.

[8]2nd Department of Oncology, Faculty of Medicine, Comenius University, and National Cancer Institute, Bratislava, Slovakia

[9]Department of Radiotherapy and Oncology, Faculty of Medicine, Pavol Jozef Šafárik University in Košice, Košice, Slovakia

[10]Department of Oncology, F. D. Roosevelt University General Hospital, Banská Bystrica, Slovakia

[11]Department of Oncology, First Faculty of Medicine, Charles University and Thomayer University Hospital, Videnska 800, 140 59 Prague, Czech Republic

[12]Department of Comprehensive Cancer Care, Masaryk Memorial Cancer Institute, Zluty kopec 7, 656 53 Brno, Czech Republic

[13]Department of Comprehensive Cancer Care, Faculty of Medicine, Masaryk University, Kamenice 5, 625 00 Brno, Czech Republic

[14]Department of Urology, Faculty of Medicine and University Hospital in Pilsen, Charles University, Czech Republic , Edvarda Beneše 1128/13, 301 00 Pilsen, Czech Republic

[15]Cancer Research Institute, Biomedical Research Center of the Slovak Academy of Sciences, Bratislava, Slovakia

# contributed equally as co-senior authors

[§] **Corresponding author**: Ondřej Fiala, M.D., Ph.D., Department of Oncology and Radiotherapeutics, Faculty of Medicine and University Hospital in Pilsen, Charles University, Czech Republic, alej Svobody 80, 304 60 Pilsen, Czech Republic. Tel.: +42 0728655488, e-mail: fialao@fnplzen.cz

**Biomarker activation score**	**Definition**	**n**	**Severe irAEs**	**Incidence (%)**
0	Neither AEC Low→High nor NLR Low→High	341	37	10.9
1	Either AEC Low→High or NLR Low→High	78	13	16.7
2	Both AEC Low→High and NLR Low→High	3	3	100.0

Abbreviations: AEC = absolute eosinophil count; NLR = neutrophil-to-lymphocyte ratio; irAE = immune-related adverse events.

Table S1. Exploratory immune biomarker activation score derived from the number of positive dynamic biomarker phenotypes.

The biomarker activation score was defined as 0 (neither AEC Low→High nor NLR Low→High phenotype), 1 (either AEC Low→High or NLR Low→High phenotype), or 2 (both AEC Low→High and NLR Low→High phenotypes).

Biomarker	Change metric	No severe irAE, median (IQR)	Severe irAE, median (IQR)	Univariable OR (95% CI)	p value	Adjusted OR (95% CI)	p value
AEC	Absolute change, per 0.1 ×10⁹/L	0.030 (−0.020 to 0.100)	0.035 (−0.028 to 0.138)	1.13 (1.03–1.24)	0.013	**1.13 (1.02–1.25)**	**0.016**
AEC	Percentage change, per 100%	24.3% (−11.0 to 100.0)	37.5% (−15.4 to 127.3)	1.04 (0.91–1.18)	0.580	1.03 (0.90–1.17)	0.700
NLR	Absolute change, per 1.0	0.324 (−0.390 to 1.192)	0.375 (−0.413 to 2.041)	1.09 (0.97–1.21)	0.136	1.10 (0.98–1.24)	0.102
NLR	Percentage change, per 100%	11.8% (−12.2 to 56.7)	16.4% (−14.1 to 57.5)	1.13 (0.83–1.53)	0.449	1.15 (0.83–1.59)	0.412

Abbreviations: AEC = absolute eosinophil count; NLR = neutrophil-to-lymphocyte ratio; irAE = immune-related adverse event; IQR = interquartile range; CI = confidence interval; OR = odds ratio.

Table S2. Continuous changes in absolute eosinophil count and neutrophil-to-lymphocyte ratio and their associations with severe immune-related adverse events.

Absolute change was calculated as the month-1 value minus the baseline value, and percentage change as 100 × (month-1 − baseline)/baseline. Odds ratios for absolute AEC change are reported per 0.1 ×10⁹/L increase, those for absolute NLR change per 1-unit increase, and those for percentage changes per 100% increase. Multivariable models were adjusted for age, sex, ECOG performance status (≥1 vs 0), IMDC poor-risk group, and treatment line (second vs third or later). Percentage AEC change was undefined for patients with a baseline AEC of zero; these patients were excluded from the corresponding analysis. Severe irAEs were defined as grade 3–4 events.

Biomarker	Threshold	Baseline cut-off	Month-1 cut-off	Low→High, n	Severe irAEs in Low→High, n (%)	Severe irAE rate in others	Adjusted OR (95% CI)	p value
AEC	Median	0.135 ×10⁹/L	0.190 ×10⁹/L	52	6 (11.5%)	12.9%	0.80 (0.32–2.00)	0.627
AEC	Upper tertile	0.200 ×10⁹/L	0.240 ×10⁹/L	38	9 (23.7%)	11.7%	2.27 (0.99–5.21)	0.053
AEC	Q4	0.230 ×10⁹/L	0.298 ×10⁹/L	43	10 (23.3%)	11.5%	**2.25 (1.02–4.97)**	**0.045**

NLR	Median	2.807	3.210	48	8 (16.7%)	11.9%	1.43 (0.63–3.29)	0.394
NLR	Upper tertile	3.488	3.998	49	11 (22.4%)	11.2%	**2.67 (1.24–5.73)**	**0.012**
NLR	Q4	4.154	4.534	42	10 (23.8%)	11.2%	**2.77 (1.25–6.17)**	**0.012**

Abbreviations: AEC = absolute eosinophil count; NLR = neutrophil-to-lymphocyte ratio; irAE = immune-related adverse event; CI = confidence interval; OR = odds ratio.

Table S3. Sensitivity analyses using alternative thresholds to define Low→High AEC and NLR phenotypes.

Low→High phenotypes were defined as transition from below the specified baseline threshold to at or above the corresponding month-1 threshold. Thresholds were derived separately from the baseline and month-1 biomarker distributions using the median, upper tertile, or upper quartile (Q4). Multivariable logistic regression models were adjusted for age, sex, ECOG performance status (≥1 vs 0), IMDC poor-risk group, and treatment line (second vs third or later). The Q4 definition represents the prespecified primary dynamic biomarker analysis; median- and upper-tertile definitions were evaluated as sensitivity analyses. AEC cut-offs are expressed in ×10^9/L; NLR is dimensionless.

Biomarker	Trajectory	n	Severe irAEs, n (%)	OR vs Low→Low (95% CI)	p value
AEC	Low→Low	273	28 (10.3%)	Reference	—
AEC	Low→High	43	10 (23.3%)	**2.65 (1.18–5.95)**	**0.018**
AEC	High→Low	44	6 (13.6%)	1.38 (0.54–3.55)	0.503
AEC	High→High	64	10 (15.6%)	1.62 (0.74–3.54)	0.225
NLR	Low→Low	276	30 (10.9%)	Reference	—
NLR	Low→High	42	10 (23.8%)	**2.56 (1.15–5.73)**	**0.022**

NLR	High→Low	42	5 (11.9%)	1.11 (0.41–3.04)	0.842
NLR	High→High	65	8 (12.3%)	1.15 (0.50–2.63)	0.740

Abbreviations: AEC = absolute eosinophil count; NLR = neutrophil-to-lymphocyte ratio; irAE = immune-related adverse event; CI = confidence interval; OR = odds ratio.

Table S4. Severe immune-related adverse event incidence and univariable associations according to four-category AEC and NLR trajectories.

Biomarker trajectories were categorized according to baseline and month-1 status relative to the respective Q4 thresholds as Low→Low, Low→High, High→Low, or High→High. Low indicates values below the respective Q4 threshold and High indicates values at or above the respective Q4 threshold. Low→Low was used as the reference category in univariable logistic regression. This exploratory sensitivity analysis was performed to determine whether the association observed with the prespecified Low→High phenotype could be explained by persistently high or other biomarker trajectories included in the primary "Others" reference group.

**Model**	**Biomarker**	**n**	**Severe irAEs**	**Firth OR (95% CI)**	**p value**
AEC model	AEC Low→High	424	54	**2.28 (1.05–4.96)**	**0.038**
NLR model	NLR Low→High	425	53	**2.78 (1.27–6.09)**	**0.011**
Combined AEC+NLR model	AEC Low→High	422	53	2.12 (0.93–4.80)	0.073
Combined AEC+NLR model	NLR Low→High	422	53	**2.80 (1.26–6.19)**	**0.011**

Abbreviations: AEC = absolute eosinophil count; NLR = neutrophil-to-lymphocyte ratio; irAE = immune-related adverse event; CI = confidence interval; OR = odds ratio.

Table S5. Firth penalized logistic regression sensitivity analyses of associations between Low→High AEC and NLR phenotypes and severe immune-related adverse events.

Firth penalized logistic regression was performed to assess the robustness of the primary estimates given the limited number of severe irAE events within the Low→High subgroups. Individual biomarker models included the respective Low→High phenotype and were adjusted for age, sex, ECOG performance status (≥1 vs 0), IMDC poor-risk group, and treatment line (second vs third or later). The combined model simultaneously included both AEC and NLR Low→High phenotypes together with the same clinical covariates.

**Characteristic**	**Complete paired AEC and NLR (n=422)**	**Incomplete paired biomarkerdata (n=69)**	**p value**
Age, years, median (IQR)	67.5 (60.9–72.9)	67.8 (60.0–73.4)	0.918
Male sex, n (%)	313 (74.2%)	47 (68.1%)	0.306
ECOG performance status ≥1, n (%)	270 (64.0%)	35 (50.7%)	**0.044**
IMDC poor risk, n (%)	50 (11.8%)	2 (2.9%)	**0.020**
Third or later nivolumab treatment line, n (%)	161 (38.2%)	26 (37.7%)	1.000
Severe grade 3–4 irAE, n (%)	53 (12.6%)	3 (4.3%)	0.063

Abbreviations: AEC = absolute eosinophil count; NLR = neutrophil-to-lymphocyte ratio; CI = confidence interval; ECOG PS = Eastern Cooperative Oncology Group Performance Status; IMDC = International Metastatic RCC Database Consortium

Table S6. Comparison of landmark-eligible patients with and without complete paired AEC and NLR measurements.

Patients in the complete-measurement group had evaluable baseline and month-1 values for both AEC and NLR. Patients with incomplete paired biomarker data fulfilled the 28-day landmark criterion and had known severe irAE status but lacked at least one measurement required for the combined longitudinal biomarker analysis. Continuous variables are presented as median (IQR) and categorical variables as n (%). Comparisons were

performed using the Mann–Whitney U test for continuous variables and Fisher's exact test for categorical variables. For consistency with the primary multivariable models, unknown ECOG and IMDC categories were coded as not ECOG ≥1 and non-poor risk, respectively.

Toxicity category	Overall n (%)	AEC Low→High n (%)	AEC others n (%)	p value	NLR Low→High n (%)	NLR others n (%)	p value
Hepatic	10 (18.9)	1 (11.1)	9 (20.5)	1.000	1 (10.0)	9 (20.9)	0.665
Pulmonary	6 (11.3)	1 (11.1)	5 (11.4)	1.000	2 (20.0)	4 (9.3)	0.315
Renal	7 (13.2)	2 (22.2)	5 (11.4)	0.588	0 (0.0)	7 (16.3)	0.323
Rheumatologic/musculoskeletal	5 (9.4)	1 (11.1)	4 (9.1)	1.000	1 (10.0)	4 (9.3)	1.000
Gastrointestinal	4 (7.5)	0 (0.0)	4 (9.1)	1.000	0 (0.0)	4 (9.3)	1.000
Neurologic	6 (11.3)	1 (11.1)	5 (11.4)	1.000	2 (20.0)	4 (9.3)	0.315
Dermatologic	4 (7.5)	2 (22.2)	2 (4.5)	0.129	2 (20.0)	2 (4.7)	0.157
Endocrine/metabolic	4 (7.5)	1 (11.1)	3 (6.8)	0.536	0 (0.0)	4 (9.3)	1.000
Hematologic	3 (5.7)	0 (0.0)	3 (6.8)	1.000	2 (20.0)	1 (2.3)	0.088
Infusion/allergic reaction	2 (3.8)	0 (0.0)	2 (4.5)	1.000	0 (0.0)	2 (4.7)	1.000
Cardiac	1 (1.9)	0 (0.0)	1 (2.3)	1.000	0 (0.0)	1 (2.3)	1.000
Ocular	1 (1.9)	0 (0.0)	1 (2.3)	1.000	0 (0.0)	1 (2.3)	1.000
Other/unspecified	2 (3.8)	0 (0.0)	2 (4.5)	1.000	0 (0.0)	2 (4.7)	1.000

Abbreviations: AEC = absolute eosinophil count; NLR = neutrophil-to-lymphocyte ratio.

Table S7. Spectrum of severe grade 3–4 immune-related adverse events (irAEs) according to early AEC and NLR dynamics in the combined biomarker cohort.

Original investigator-reported toxicity terms were harmonized into standardized organ-system categories before analysis. Patients experiencing more than one severe irAE contributed to each relevant toxicity category; therefore, the sum of individual toxicity categories exceeds the total number of patients with severe irAEs. Comparisons between biomarker-defined subgroups were performed using Fisher's exact test. The combined biomarker cohort comprised patients with complete data for both AEC and NLR dynamic analyses who fulfilled the prespecified 28-day landmark criteria (n = 422).

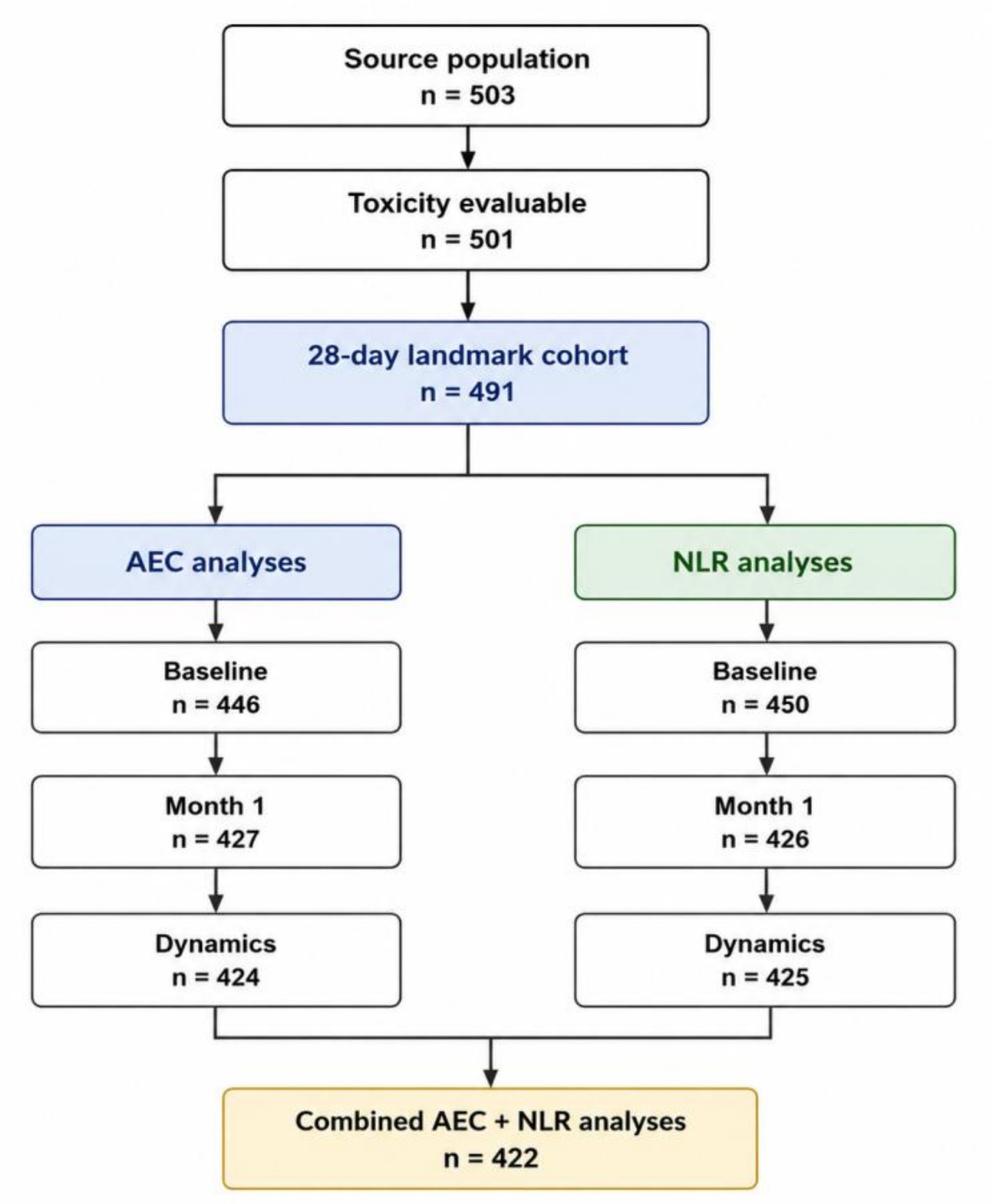

Figure S1. Study flow diagram illustrating derivation of the landmark biomarker cohort and evaluable analytical subsets.

Individual biomarker analyses were performed in evaluable subsets according to the availability of baseline and/or month-1 laboratory measurements (complete-case analysis).

Abbreviations: AEC, absolute eosinophil count; NLR, neutrophil-to-lymphocyte ratio.
